# Caveolin-1 Regulates P2Y_2_ Receptor Signaling during Mechanical Injury in Human 1321N1 Astrocytoma

**DOI:** 10.3390/biom9100622

**Published:** 2019-10-18

**Authors:** Magdiel Martínez, Namyr A. Martínez, Jorge D. Miranda, Héctor M. Maldonado, Walter I. Silva Ortiz

**Affiliations:** 1 Physiology Department, University of Puerto Rico, School of Medicine, San Juan 00936-5067, Puerto Rico; 2 Pharmacology Department, Universidad Central del Caribe, Bayamón 00960-6032, Puerto Rico

**Keywords:** caveolin-1, P2Y2, signaling, caveolae, brain injury, astrocytoma

## Abstract

Caveolae-associated protein caveolin-1 (Cav-1) plays key roles in cellular processes such as mechanosensing, receptor coupling to signaling pathways, cell growth, apoptosis, and cancer. In 1321N1 astrocytoma cells Cav-1 interacts with the P2Y_2_ receptor (P2Y_2_R) to modulate its downstream signaling. P2Y_2_R and its signaling machinery also mediate pro-survival actions after mechanical injury. This study determines if Cav-1 knockdown (KD) affects P2Y_2_R signaling and its pro-survival actions in the 1321N1 astrocytoma cells mechanical injury model system. KD of Cav-1 decreased its expression in 1321N1 cells devoid of or expressing hHAP2Y_2_R by ~88% and ~85%, respectively. Cav-1 KD had no significant impact on P2Y_2_R expression. Post-injury densitometric analysis of pERK1/2 and Akt activities in Cav-1-positive 1321N1 cells (devoid of or expressing a hHAP2Y_2_R) revealed a P2Y2R-dependent temporal increase in both kinases. These temporal increases in pERK1/2 and pAkt were significantly decreased in Cav-1 KD 1321N1 (devoid of or expressing a hHAP2Y_2_R). Cav-1 KD led to an ~2.0-fold and ~2.4-fold decrease in the magnitude of the hHAP2Y_2_R-mediated pERK1/2 and pAkt kinases’ activity, respectively. These early-onset hHAP2Y_2_R-mediated signaling responses in Cav-1-expressing and Cav-1 KD 1321N1 correlated with changes in cell viability (via a resazurin-based method) and apoptosis (via caspase-9 expression). In Cav-1-positive 1321N1 cells, expression of hHAP2Y_2_R led to a significant increase in cell viability and decreased apoptotic (caspase-9) activity after mechanical injury. In contrast, hHAP2Y_2_R-elicited changes in viability and apoptotic (caspase-9) activity were decreased after mechanical injury in Cav-1 KD 1321N1 cells expressing hHAP2Y_2_R. These findings support the importance of Cav-1 in modulating P2Y_2_R signaling during mechanical injury and its protective actions in a human astrocytoma cell line, whilst shedding light on potential new venues for brain injury or trauma interventions.

## 1. Introduction

Traumatic injury, stress, and damage to the cells of the central nervous system (CNS) lead to the release of neurotransmitters, growth factors, and pro-inflammatory cytokines [1]. Among such factors, nucleotides have gained prominence as key modulators of gliotic responses after brain trauma [2,3]. It has been shown that ATP and UTP (via interaction with P2 receptors) can promote astrocyte stellation, proliferation, and migration, and can modulate the expression of glial fibrillary acidic protein (GFAP), thus contributing to the process of reactive astrocytosis [4,5,6]. The P2Y_2_R has been shown to play an important protective role as an inducer of neurite outgrowth and a promoter of cell survival mechanisms [7,8,9]. Functional and stable expression of the P2Y_2_R in 1321N1 human astrocytoma cells, in contrast to normally P2-devoid 1321N1 cells, provides an anti-apoptotic effect by activating pro-survival signaling pathways after in vitro mechanical injury [10,11].

Caveolin-1 (Cav-1), one of the main raft scaffolding proteins [12,13], has been shown to modulate multiple cellular responses by coupling membrane receptors to downstream signaling molecules [14]. In this context, we have recently shown that P2Y_2_R resides in Cav-1 raft microdomains of 1321N1 cells, and their interaction regulates P2Y_2_R signal transduction by extracellular ATP, including intracellular calcium mobilization, as well as Akt and ERK1/2 activation [15]. Therefore, the interaction between P2Y_2_R and Cav-1 in raft microdomains is a key factor mediating nucleotide signaling in astrocytic cells. This study was undertaken to assess if Cav-1 expression in 1321N1 astrocytoma cells affects P2Y_2_R signaling and pro-survival actions after mechanical injury. Our results demonstrate that Cav-1 modulates P2Y_2_R signaling and pro-survival actions in the 1321N1 mechanical injury model system. Therefore, Cav-1 and P2Y_2_R interaction is a key factor mediating nucleotide signaling in astrocytic cells regulating their glioprotective functions in the CNS. Understanding the mechanisms of purinergic receptor signaling in astrocytic processes may provide novel targets for therapeutic strategies in the management of brain injury, as well as other neurological conditions.

## 2. Materials and Methods

### 2.1. Antibodies and Reagents

The following antibodies and reagents were used in this study: anti-phospho-Akt (Ser473) (D9E) (1:2000), anti-Akt (pan) (C67E7) (1:1000), anti-phospho-p44/42 MAPK (ERK1/2) (Thr202/Tyr204, D13.14.4E) (1:2000), and anti-total p44/42 MAPK (ERK1/2) (1:1000) antibodies from Cell Signaling Technology (Boston, MA, USA); rabbit polyclonal anti-caveolin-1 (1:7500), anti-GAPDH (1:10,000) from Sigma-Aldrich (St. Louis, MO, USA); anti-rabbit IgG-Peroxidase antibody and resazurin sodium salt were obtained from Sigma-Aldrich (St. Louis, MO, USA); suramin hexasodium salt was obtained from Tocris Bioscience (Ellisville, MO, USA). Control (SC108080) and human caveolin-1 (SC29241) shRNA lentiviral particles were purchased from Santa Cruz Biotechnology (Santa Cruz, CA, USA). All other reagents, unless mentioned, were obtained from Sigma-Aldrich (St. Louis, MO, USA).

### 2.2. Cell Culture

Wild-type (WT) human 1321N1 astrocytoma cells devoid of functional P2 receptors and human N-terminal HA-tagged P2Y2R (hHAP2Y_2_R) expressing 1321N1 astrocytoma cells were a kind gift from Dr. Gary A. Weisman, University of Missouri. WT human 1321N1 astrocytoma cells were grown at 37 °C in a humidified 5% CO_2_ atmosphere in Dulbecco’s modified Eagle’s medium (DMEM) supplemented with 5% (*v/v*) FBS (Life Technologies Corp., Carlsbad, CA, USA), 100 U/mL penicillin, and 100 mg/mL streptomycin. Human 1321N1 cells expressing hHAP2Y_2_R (hHAP2Y_2_R 1321N1) were grown as above, but in the presence of 500 μg/mL G418, and cells that were infected with Cav-1 or scrambled shRNA-containing lentiviral particles in the presence of 5 μg/mL of puromycin dihydrochloride.

### 2.3. Mechanical Injury

shRNA Lentiviral Infection–Cells were infected with either control shRNA lentiviral particles (scrambled or non-targeted shRNA) or caveolin-1 shRNA-containing lentiviral particles obtained from Santa Cruz Biotechnology (Santa Cruz, CA, USA), following the manufacturer’s recommendations. After infection, stable cell clones expressing the shRNA constructs were isolated.

### 2.4. Mechanical Injury

1321N1 astrocytoma cells were seeded at a concentration of 8.0 × 10^5^ in collagen-coated Flex Plates (FlexCell International Corp., Hillbrough, NC, USA) and incubated with DMEM containing 5% (*v/v*) FBS. After 24 h, cells were incubated in serum-free medium for 16–24 h prior to being subjected to mechanical injury. Before each experiment, the injury controller device was calibrated as described by the manufacturer (Custom Design & Fabrication, Richmond, VA, USA) [4,10]. Briefly, the delay was set to 50 ms on the cell injury controller device and the regulator pressure was set to 15 psi [4]. The cell injury controller trigger was pressed a couple of times until the peak pressure became stable. With one hand the adapter plug was set firmly over the well plate, while the other hand pushed the trigger. The cell injury controller regulator was set to a well peak injury pressure from 3.5 to 4.5 psi, equivalent to a severe injury level (155% membrane stretch) [4]. For time course experiments, cells were subjected to mechanical strain by means of a Cell Injury Controller II (Custom Design & Fabrication, Richmond, VA, USA) and returned to the incubator at 37 °C for 5, 10, 15, 30 and 60 min [4,10]. For viability and caspase-9 measurement experiments, cells were treated as above but returned to the incubator for 24 h. Uninjured cells in wells of collagen coated Flex Plates served as controls.

### 2.5. Cell Viability Assay

Cell viability assay was carried out using resazurin salt assay according to the manufacturer’s instructions. Briefly, after mechanical injury the cells (uninjured control and injured) were returned to the incubator for 24 h. Then, the medium was removed and 2 mL of a resazurin solution (20 μg/mL solution) prepared in fresh medium was added to each well. Following 3 h of incubation, samples of 200 μL were taken and added to a 96-well plate in triplicate. AB fluorescence was quantified at the respective excitation and emission wavelengths of 540 and 595 nm using a Tecan Infinite M200 Pro microplate reader (Tecan, Männedorf, Switzerland). The results were averaged over four different independent experiments. For each experiment, control/background wells containing only the AB solution without cells were also prepared and incubated for 3 h. Percent of cell viability was expressed as the injured relative fluorescence units (RFU) over the uninjured control RFU * 100.

### 2.6. Caspase-9 Activity

Caspase activity within the injured cells was determined using a commercial fluorimetric assay kit (R&D System). Briefly, control and injured cells were lysed and centrifuged at 17,000× *g* for 20 min to collect the supernatant. The assay was conducted in a flat-bottom 96-well microplate. To each sample containing cell lysate (100 μg of protein in 50 μL) and 50 μL of 2× reaction buffer, 5 μL of caspase-9 fluorometric substrate (LEHD-AFC) were added. The reaction was incubated for 2 h at 37 °C and then fluorescence, which is indicative of caspase activation, was determined at 400 nm excitation and emission at 505 nm on a fluorescent plate reader (Tecan Infinite M200, Tecan, Männedorf, Switzerland). Percent of caspase-9 activity was expressed as the injured RFU over the uninjured control RFU * 100.

### 2.7. Protein Extraction

Cells were washed with ice-cold PBS and lysed with CelLytic™ Mammalian Cell Lysis/Extraction Reagent (Sigma, Saint Louis, MO, USA), supplemented with 1% protease cocktail with phosphatase inhibitor, as above. Extracts were maintained with constant agitation for 30 min at 4 °C and then centrifuged for 20 min at 17,000× *g*. Supernatants were collected and used to determine total protein concentration using the Bradford Quick-Start Protein Assay (Bio-Rad Laboratories, Hercules, CA, USA).

### 2.8. SDS-PAGE and Immunoblot Analysis

SDS-PAGE and immunoblots were performed as previously described [15] with modifications. Equal amounts of whole-cell protein extracts were suspended in 6x Laemmli Sample Buffer (0.375 M Tris pH 6.8, 12% (*w/v*) SDS, 60% (*v/v*) glycerol, 0.6 M DTT, 0.06% (*w/v*) bromophenol blue) then heated and electrophoresed on Pre-Cast TGX-SDS gels (Bio-Rad Laboratories, Hercules, CA, USA). Proteins in the gel were transferred to nitrocellulose membranes using the Bio-Rad Turbo Trans-Blot apparatus at preprogrammed recommended settings. Membranes were blocked with 5% (*w/v*) non-fat milk in TBST (25 mM Tris-HCl, pH 7.4, 150 mM NaCl, and 0.1% (*v/v*) Tween-20) for 1 h at 22 °C and the appropriate primary antibodies were added overnight at 4 °C. Membranes were then washed with TBST and probed with the corresponding horseradish peroxidase-conjugated IgG secondary antibody (1:18,000) at 22 °C for 1 h. Membranes were washed several times with TBST and blots were developed using an ECL kit (SuperSignal Femto, Pierce, Rockford, IL, USA). All images were obtained using a Bio-Rad VersaDoc 4000 System (Bio-Rad Laboratories, Hercules, CA, USA), as previously described [15] and densitometric analysis was done using NIH ImageJ software. To reprobe with different antibodies, the membranes were first stripped using Restore PLUS Western Blot Stripping Buffer (Thermo Scientific, Waltham, MA, USA) at 22 °C for 30 min, washed extensively, reblocked with 5% (*w/v*) non-fat milk in TBST, and then incubated with the appropriate antibodies.

### 2.9. Statistical Analysis

**Figure 1:**
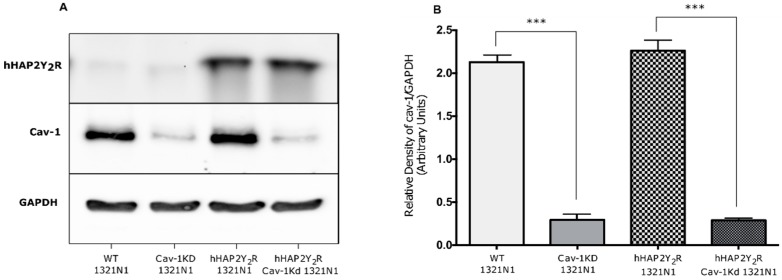
shRNA-mediated knockdown of caveolin-1 expression of 1321N1 astrocytoma cells. (**A**) Immunoblot analysis of hHAP2Y_2_R, caveolin-1 (Cav-1), and GAPDH (control) expression in serum-starved wild-type (WT) 1321N1 cells (lane 1), human 1321N1 cells expressing hHAP2Y_2_R (hHAP2Y_2_R 1321N1 cells) (lane 3), or cells infected with Cav-1 shRNA lentiviral particles (lanes 2 and 4; Cav-1 knockdown (KD)). (**B**) Densitometric analysis of immunoblots indicates the level of Cav-1 normalized to GAPDH expression. Results are presented as the means ± S.E.M. (n = 3; *** *p* < 0.001 as determined by one-way ANOVA).

**Figure 2:**
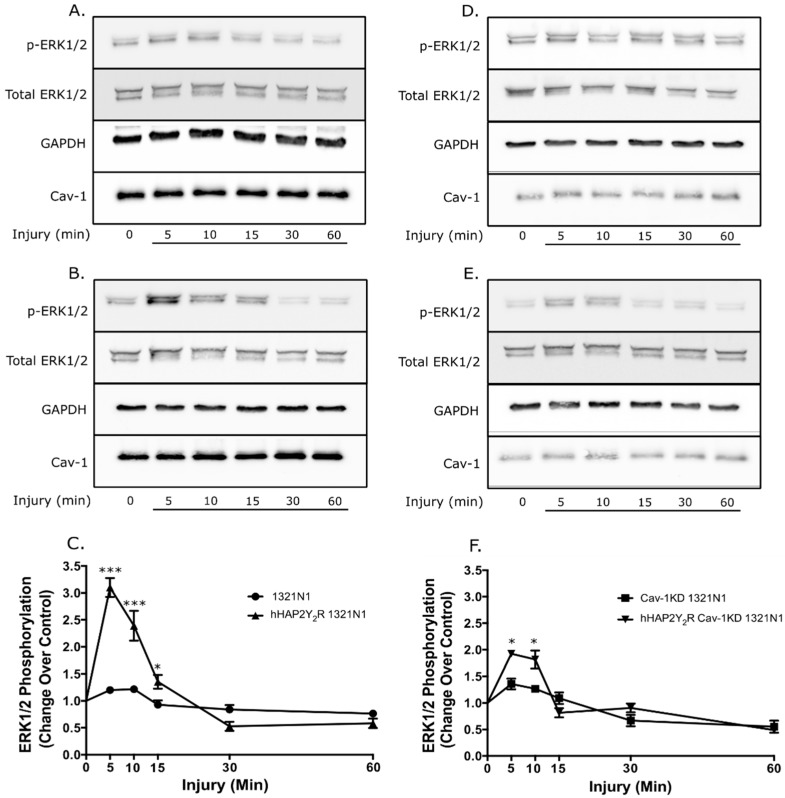
Cav-1 KD reduced the P2Y_2_R-mediated ERK1/2 phosphorylation after mechanical injury. Post-injury ERK1/2 phosphorylation time course of (**A**–**C**) Cav-1-expressing and (**D**–**F**) Cav-1 KD 1321N1 cell lines. Immunoblots for (**A**) Cav-1-expressing WT-1321N1 and (**B**) Cav-1/hHAP2Y_2_R-expressing 1321N1 cells. Densitometric analysis of the latter immunoblots is shown in (**C**), revealing the post-injury hHAP2Y_2_R-mediated increased ERK1/2 activity. Immunoblots for (**D**) Cav-1 KD WT-1321N1 and (**E**) Cav-1 KD/hHAP2Y_2_R-expressing 1321N1 cells. Densitometric analysis of the latter immunoblots is shown in (**F**), revealing the diminished post-injury hHAP2Y_2_R-mediated increased ERK1/2 activity in Cav-1KD 1321N1 cells. Cells were subjected to a severe traumatic injury and immunoblot analysis was done as described in the Materials and Methods section. ERK1/2 phosphorylation and total ERK1/2, Cav-1, and GAPDH (control) expression in equal amounts of protein were determined by Western blot analysis. Immunoblots are representative of at least three independent experiments. In (C) and (F), ERK1/2 phosphorylation was normalized using the formula: phosphorylated ERK1/2/(total ERK1/2 + GAPDH) and expressed as a percentage of untreated controls at 0 min. Values represent the means ± S.E.M. (n = 4), * *p* < 0.05 and *** *p* < 0.001 (one-way ANOVA) represent statistically significant differences between P2Y_2_R-devoid and P2Y_2_R-expressing 1321N1 cells.

Mean densitometric values of treatment data from at least four independent experiments were calculated and expressed as a percentage of mean values from untreated controls (which were set to 100%). One-way ANOVA followed by multiple Tukey comparison post-test or unpaired Student’s *t*-test were used for comparison of multiple groups or two groups, respectively. A *p*-value less than 0.05 between control and experimental groups was considered statistically significant. All analyses were performed using GraphPad Prism, version 7.0e for Mac OS X (GraphPad Software Inc, San Diego, CA, USA). Parameter data were expressed as mean ± S.E.M. of at least four independent experiments and were subjected to unpaired Student’s *t*-test. Differences between mean values were considered significant when *p* < 0.05. To determine the relative degree of P2Y_2_R-mediated changes in ERK1/2 and Akt signaling in the various 1321N1 cell lines, the pharmacokinetic parameter of area under the curve (AUC) (using the GraphPad Prism total peak area parameter) was estimated from the post-injury time course experiments shown in Figure 1 and Figure 2. Values represent AUC means ± S.E.M. (n = 4), where ** *p* < 0.01.

## 3. Results

### 3.1. Cav-1 Knockdown Modulates P2Y2R Signaling Behavior after Mechanical Injury

**Figure 3:**
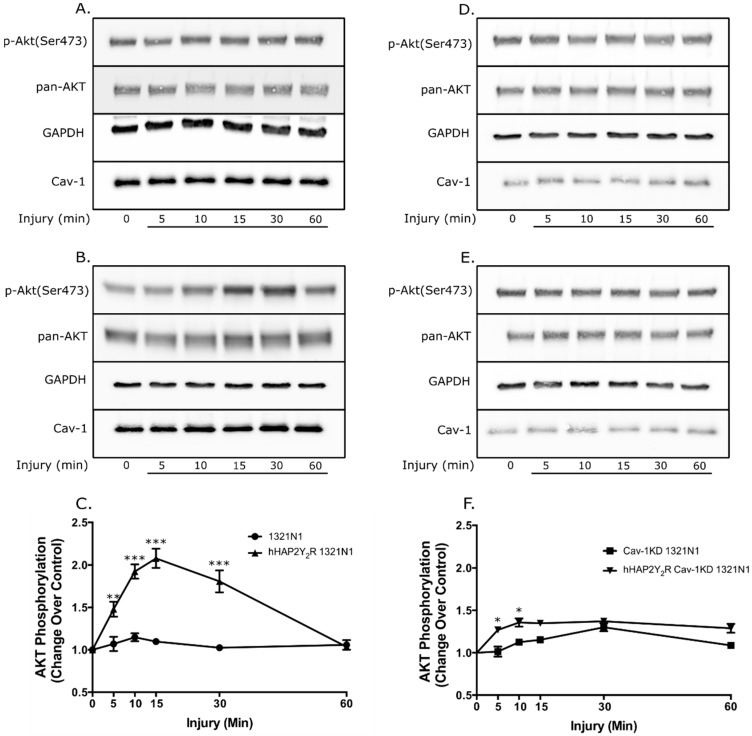
Cav-1 KD reduced the P2Y_2_R-mediated Akt phosphorylation after mechanical injury. Post-injury Akt phosphorylation time course of (**A**–**C**) Cav-1-expressing and (**D**–**F**) Cav-1 KD 1321N1 cell lines. Immunoblots for (**A**) Cav-1-expressing WT-1321N1 and (**B**) Cav-1/hHAP2Y_2_R-expressing 1321N1 cells. Densitometric analysis of the latter immunoblots is shown in (**C**), revealing the post-injury hHAP2Y_2_R-mediated increased Akt activity. Immunoblots for (**D**) Cav-1 KD WT-1321N1 and (**E**) Cav-1 KD/hHAP2Y_2_R-expressing 1321N1 cells. Densitometric analysis of the latter immunoblots is shown in (**F**), revealing the diminished post-injury hHAP2Y2R-mediated increased Akt activity in Cav-1 KD 1321N1 cells. Cells were lysed and Akt phosphorylation on Ser473 and total Akt, Cav-1, and GAPDH (control) expression in equal amounts of protein were determined by Western blot analysis. Immunoblots are representative of at least three independent experiments. In panels C and F, Akt phosphorylation on Ser473 was normalized using the formula: phosphorylated Akt/(pan Akt + GAPDH) and expressed as a percentage of untreated controls at 0 min. Values represent the means ± S.E.M. (n = 4), where * *p* < 0.05, ** *p* < 0.01, and *** *p* < 0.001 (one-way ANOVA) represent statistically significant differences between P2Y_2_R-devoid and P2Y_2_R-expressing 1321N1 cells.

To further establish the pathophysiological relevance of the Cav-1–P2Y_2_R interaction, we determined if caveolin-1 expression in 1321N1 astrocytoma cells impacted the P2Y_2_R signaling responses (ERK 1/2 and Akt) after mechanical injury. Figure 1 demonstrates the effective knockdown (KD) of Cav-1 and stable expression of the hHAP2Y_2_R. Knockdown of Cav-1 in 1321N1 cells devoid of or expressing a hHAP2Y_2_R resulted in ~88% and ~85% decrease in expression, respectively. In turn, Cav-1 KD did not have a significant impact on the hHAP2Y2R expression. Similarly, under our experimental conditions, no significant differences in basal ERK1/2 and Akt were seen between the different 1321N1 astrocytoma cell lines, including the Cav-1 KD clones (Supplementary Figure S1). In turn, Figure 2 shows that after mechanical injury there was an increased ERK1/2 activity in Cav-1-expressing 1321N1 cells that also express the hHAP2Y2R, in contrast to P2Y_2_R-devoid cells (WT-1321N1 cells) (Figure 2A–C). Yet, Cav-1 KD led to a significantly reduced P2Y_2_R-mediated ERK1/2 phosphorylation after mechanical injury (Figure 2D–F). Figure 3 also shows that Akt signaling was similarly affected. As previously established, P2Y_2_R expression in 1321N1 astrocytoma cells caused an increased time-dependent Akt phosphorylation after mechanical injury (Figure 3A–C). Meanwhile, Cav-1 KD significantly reduced P2Y_2_R-mediated Akt phosphorylation at Ser473 after mechanical injury in 1321N1 cells (Figure 3D–F). The selectivity of the P2Y2R-mediated changes in ERK1/2 and Akt was further validated by use of the antagonist suramin (Supplementary Figure S2).

The magnitude and significance of the Cav-1 KD-elicited changes in P2Y_2_R-mediated ERK1/2 and Akt signaling post mechanical injury are summarized in Table 1. Analysis of the post-injury phosphorylation time-course data contained in Figure 2 and Figure 3 was done using the pharmacokinetic parameter of AUC (see Materials and Methods). The analysis revealed a temporal increase in both kinases’ activities endowed by expression of the hHAP2Y_2_R (net pERK1/2 17.98 (+/−2.284); net pAkt 31.73 (+/−3.51)). In contrast, the magnitudes of these temporal increases in pERK1/2 and pAkt were significantly decreased in Cav-1 KD 1321N1 cells (devoid of or expressing a hHAP2Y_2_R) (net pERK1/2 5.94 (+/−0.90); net pAkt 9.35 (+/−1.13)). Hence, Cav-1 KD led to a ~2.0-fold and ~2.4-fold decrease in the magnitude of the P2Y_2_R-mediated pERK1/2 and pAkt kinases’ activity, respectively. In addition, analysis of both ERK1/2 and Akt activity in 1321N1 cells devoid of the P_2_Y2R revealed that Cav-1 KD did not elicit a statistically significant difference when compared to the WT 1321N1 (Table 1). In contrast, analysis of P2Y_2_R-expressing 1321N1 cells showed a significant reduction in both ERK1/2 and Akt activity elicited by Cav-1KD (*p* < 0.001).

### 3.2. Knockdown of Caveolin-1 Expression Inhibits hP2Y_2_R-Mediated Increased Cell Viability and Anti-Apoptotic Actions after Mechanical Injury

**Figure 4:**
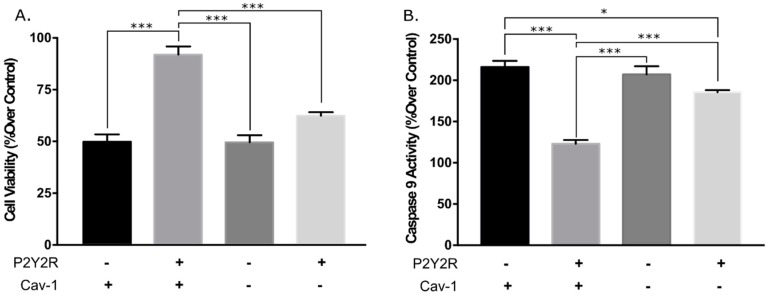
Cav-1 KD inhibits the (**A**) P2Y_2_R-mediated increased cell viability and (**B**) anti-apoptotic action after mechanical injury. The relative expression of both P2Y_2_R and Cav-1 are indicated on the *x*-axes of panels (**A**) and (**B**). After injury, cell cultures were returned to the incubator and further incubated for 24 h. Cell viability and caspase-9 activity were measured using AB and caspase-9 fluorometric kit, as described in the Materials and Methods section. Uninjured cells in wells of Flex Plates served as controls. Values are mean ± S.E.M. (n = 4) expressed as a percentage of responses in non-injured cells (upper panel). *** *p* < 0.001, * *p* < 0.05 (one-way ANOVA).

It has been shown that P2Y_2_R signaling responses in 1321N1 astrocytoma cells mediate its cell survival and anti-apoptotic action upon mechanical injury [10]. To further investigate the role of Cav-1 in the P2Y_2_R-mediated pro-survival and antiapoptotic actions, the aforementioned 1321N1 astrocytoma cell lines were subjected to mechanical injury, and cell viability and caspase-9 activity were measured 24 h post injury. Results showed that in Cav-1-positive 1321N1 cells the expression of the hHAP2Y_2_R led to a significant increase in cell viability of ~92%, compared with ~49% in WT (P2Y_2_R devoid) 1321N1 cells (Figure 4A). In turn, Cav-1 KD significantly diminished the P2Y2R-elicited cell viability increase after mechanical injury from ~92% to ~62% (Figure 3A). Moreover, in Cav-1-positive 1321N1 cells the expression of the P2Y_2_R exerted an anti-apoptotic action as revealed by a significant decrease in caspase-9 activity from ~216% to ~123%. This anti-apoptotic action (or decreased caspase-9 activity) was significantly diminished in Cav-1 KD expressing the P2Y_2_R in 1321N1 cells (Figure 4B). Collectively, the data demonstrate that Cav-1 is necessary for the P2Y_2_R-mediated signaling, pro-survival, and anti-apoptotic actions after mechanical injury in human 1321N astrocytoma cells. Hence, these diminished pathophysiological responses are due to the diminished cell signaling of the P2Y_2_R after diminished Cav-1 expression.

## 4. Discussion

Traumatic brain injury (TBI) caused by accidents, violence, or contact sports injuries (i.e., concussions) affects millions of people around the world, demands billions of dollars from medical care systems, and can lead to significant individual permanent or transitory disabilities [16,17,18]. During TBI, extracellular nucleotides originated from damaged cells are released [19,20,21], leading to nucleotide receptor(s) activation, including the P2Y_2_R. Stimulation of the P2Y_2_R leads to the activation of multiple signal transduction pathways [4,9,10,22] that are important mediators of cell survival [3,10] metabolism control, cell cycle progression [23,24,25] and differentiation, as well as inflammation and apoptosis [3,10,22,24]. The P2Y_2_R has been shown to play an important protective role as an inducer of neurite outgrowth and a promoter of cell survival mechanisms [8,9,21,25]. Functional and stable expression of the hP2Y_2_R in 1321N1 human astrocytoma cells, in contrast to normally P2-devoid 1321N1 cells, provides an anti-apoptotic effect by activating pro-survival signaling pathways after in vitro mechanical injury [10]. Similarly, Cav-1 plays a pivotal role in cell proliferation and resistance to apoptosis, as well as protection of neurons from hypoxic injury, via the ERK1/2 signaling pathway [26,27,28,29,30,31].

**Figure 5:**
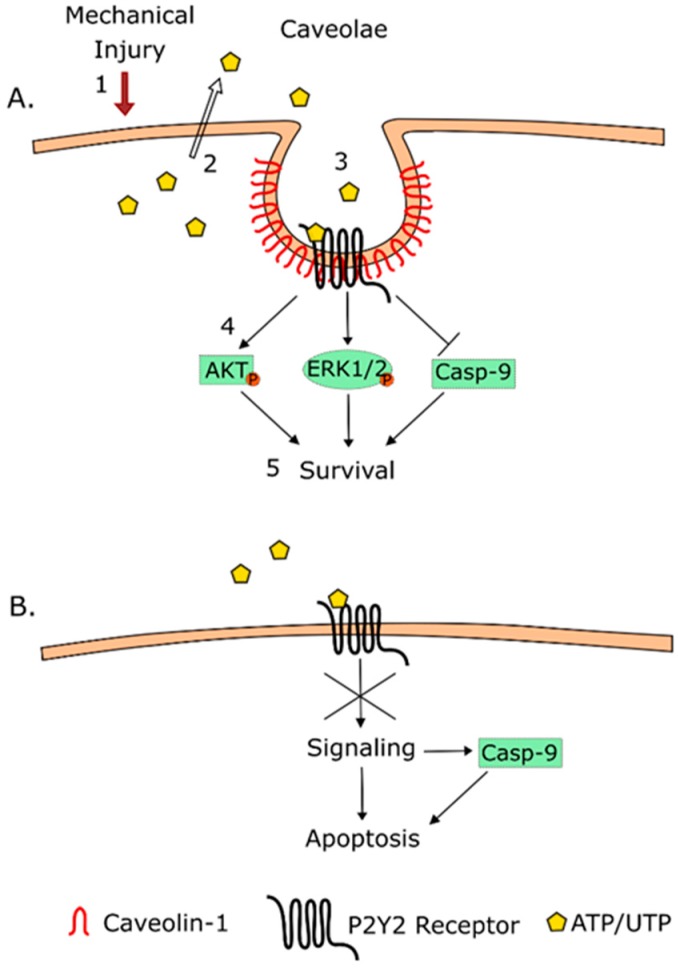
Caveolin-1 is necessary for the P2Y_2_R anti-apoptotic action in 1321N1 cells after mechanical injury. A schematic representation describing the Cav-1-dependent P2Y_2_R-mediated signaling pathways investigated in this study. (**A**) Nucleotide agonists’ activation of the P2Y_2_R signaling pathways and subsequent antiapoptotic action during mechanical injury. (**B**) Caveolin-1 knockdown leads to P2Y_2_R uncoupling from its signaling pathways.

In this context, we recently established that P2Y_2_R resides in Cav-1 raft microdomains and their interaction regulates P2Y_2_R signal transduction by extracellular ATP, including intracellular calcium mobilization and Akt and ERK1/2 activities [15]. The latter findings strongly suggest that the interaction between P2Y_2_R and Cav-1 in raft microdomains is a key factor mediating nucleotide signaling in astrocytic cells regulating their protective, trophic, and degenerative functions in the CNS. The present study demonstrates that Cav-1 knockdown modulated P2Y_2_R signaling behavior after mechanical injury (Figure 2 and Figure 3, and Table 1). The post-mechanical injury P2Y_2_R-mediated increased ERK1/2 activity in Cav-1-expressing 1321N1 cells was significantly decreased by Cav-1 KD (Figure 2 and Table 1). Similarly, post-mechanical injury P2Y_2_R-mediated increased Akt activity in Cav-1-expressing 1321N1 cells was significantly decreased by Cav-1 KD (Figure 3 and Table 1). These P2Y_2_R-mediated increased signaling responses are required for its pro-survival and anti-apoptotic actions. Accordingly, the present study demonstrates that knockdown of Cav-1 expression in 1321N1 cells also inhibited P2Y_2_R-mediated increased cell viability and anti-apoptotic actions after mechanical injury (Figure 5). Altogether, these findings further support the physiological and pathophysiological relevance of the Cav-1–P2Y_2_R interaction in this injury glial cell model system.

The findings of the present study are depicted in Figure 5. Yet, the exact mechanisms by which caveolin-1 regulates P2Y_2_R-mediated Akt and ERK1/2 phosphorylation post injury remain to be elucidated. In this context, both P2Y_2_R and Cav-1 trafficking, interactions with alternative scaffolding molecules (flotillin, cofilin, arrestins, among others), and pharmacodynamic processes (i.e., desensitization) deserve consideration [32]. Most importantly, analysis of the Cav-1–P2Y_2_R protein-protein interaction domains may unveil promising targets for drug discovery and the development of treatment for conditions where both have been shown to play a role, such as neurodegenerative diseases [19,20], cancer [25], and brain injury [2,3,21,23,33].

## Figures and Tables

**Table 1:** Pharmacokinetic (area under the curve—AUC) analysis of P2Y_2_R-mediated signaling responses in 1321N1 cells post injury ^†^.

	CAV-1-EXPRESSING			CAV1 KD		
SIGNALING	WT-1321N1	hHAP2Y_2_R 1321N1	*p*-Value	CAV-1 KD 1321N1	CAV-1 KD hHAP2Y_2_R 1321N1	*p*-Value
**pERK1/2**	2.33 (+/−0.48)	20.31 (+/−2.24)	***	2.47 (+/−0.65)	8.41 (+/−0.95)	*
**pAkt**	5.50 (+/−1.61)	37.23 (+/−2.81)	***	8.59 (+/−1.54)	17.93 (+/−1.50)	*
**Net P2Y2R-mediated pERK1/2 signaling**		17.98 (+/−2.284)			5.94 (+/−0.90)	**
**Net P2Y2R-mediated pAkt signaling**		31.73 (+/−3.51)			9.35 (+/−1.13)	***

^†^ To determine the relative degree of P2Y_2_R-mediated changes in ERK1/2 and Akt signaling in the various 1321N1 cell lines, the pharmacokinetic parameter area under the curve (AUC) (using the GraphPad Prism total peak area parameter) was estimated from the post-injury time course experiments shown in Figure 2 and Figure 3. Values represent the means ± S.E.M. (n = 4), where * *p* < 0.05, ** *p* < 0.01, and *** *p* < 0.001.

## Supplementary Materials

The following are available online at https://www.mdpi.com/2218-273X/9/10/622/s1, Figure S1: 1321N1 astrocytoma cell line clones pAKT and pERK1/2 basal activity; Figure S2: Inhibition of P2Y_2_R-mediated ERK1/2 and AKT phosphorylation by suramin after mechanical injury.
