# Supplementary material for: Caveolin-1 Regulates P2Y2 Receptor Signaling during Mechanical Injury in Human 1321N1 Astrocytoma
Source: Biomolecules. 2019 Oct 18;9(10):622. doi: 10.3390/biom9100622 (PMC6843573; doi:10.3390/biom9100622)
Supplement: Supplementary file 1 [file biomolecules-09-00622-s001.pdf]

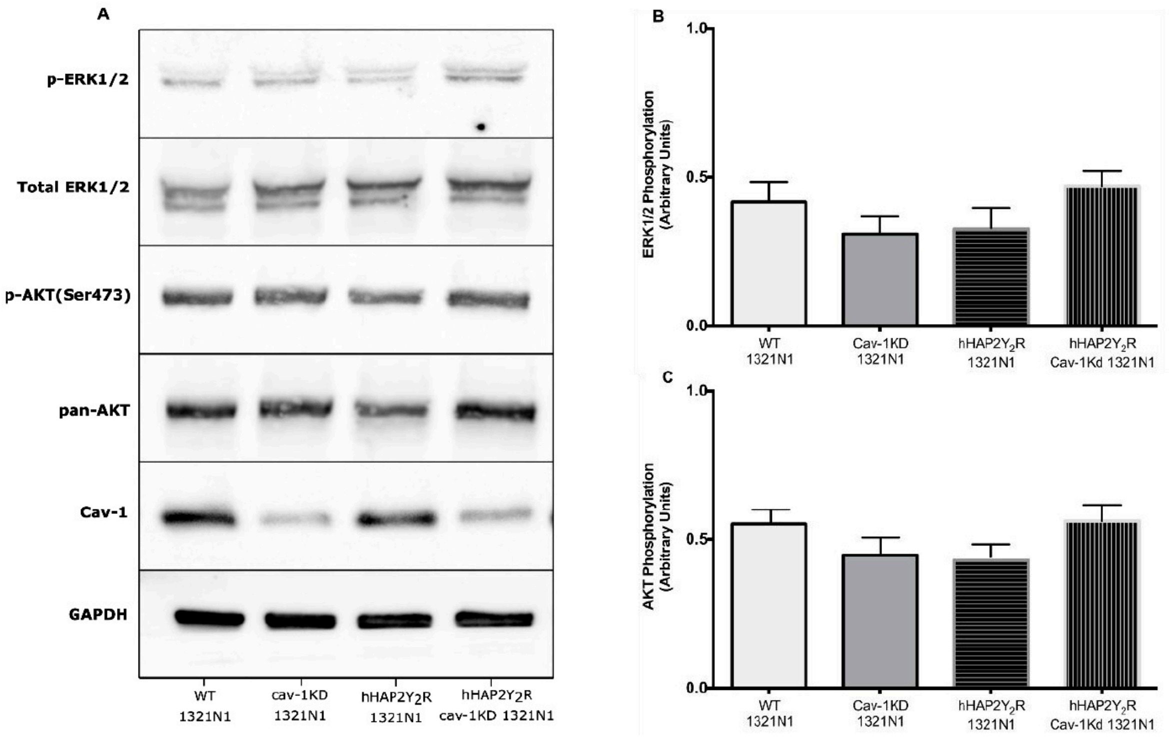

Figure **S1.** 1321N1 astrocytoma cell line clones pAKT and pERK1/2 basal activity. Immunoblots for pAKT (Ser473) and pERK1/2 (Panel A) for Cav-1-expressing and Cav-1 KD cells. Densitometric analysis of the latter immunoblots is shown in Panel B and Panel C revealing no difference in the pAKR(Ser473) and pERK1/2 basal activity. Immunoblot analysis was done as described in 'Materials and Methods'. pAKT, total AKT, ERK1/2 phosphorylation and total ERK1/2, Cav-1 and GAPDH (control) expression in equal amounts of protein were determined by Western blot analysis. Immunoblots are representative of at least three independent experiments. In Panels B and C, AKT and ERK1/2 phosphorylation was normalized using the formula: phosphorylated phopho-protein / (total protein + GAPDH) and expressed as a percentage of untreated controls at 0 min. Values represent the means ± S.E.M. (n = 4).

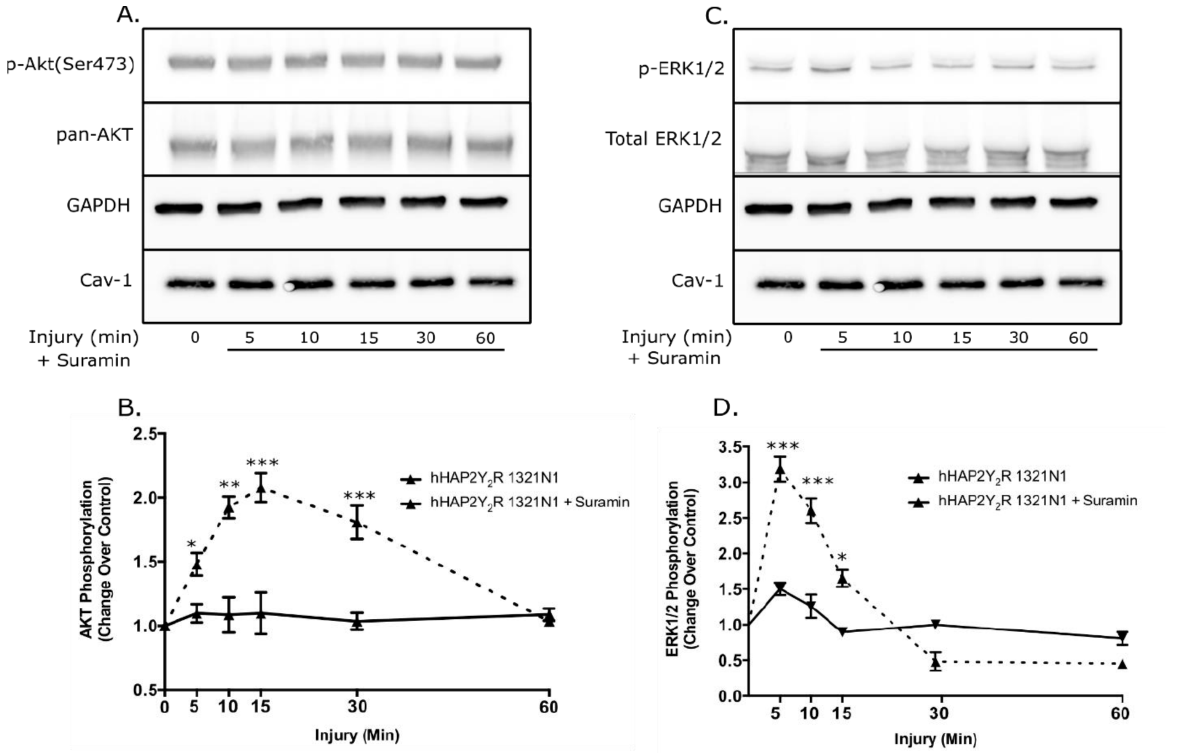

Figure S2. Inhibition of P2Y2R-mediated ERK1/2 and AKT phosphorylation by suramin after mechanical injury. hHAP2Y2R 1321N1 astrocytoma cells were subjected to traumatic injury as described in 'Materials and methods". Cell cultures were treated with 100 µmol/L suramin for 30 min before injury. Immunoblots for pAKT (Ser473) (Panel A) and pERK1/2 (Panel C) activation after injury. Densitometric analysis of the latter immunoblots is shown in Panel B and Panel D, reveal the suramin inhibition of post-injury hHAP2Y2R mediated activity in pAKT (Ser473) and pERK1/2. Doted line in Panel B and Panel D is from data in Figure 2 and 3. Experimentes were performed in parallel. Immunoblot analysis was done as described in 'Materials and Methods'. pAKT, total AKT, ERK1/2 phosphorylation, total ERK1/2, Cav-1 and GAPDH (control) expression in equal amounts of protein were determined by Western blot analysis. Immunoblots are representative of at least three independent experiments. In Panels B and D, AKT and ERK1/2 phosphorylation was normalized using the formula: phosphorylated phopho-protein / (total protein + GAPDH) and expressed as a percentage of untreated controls at 0 min. Values represent the means ± S.E.M. (n = 4).
